# A stepwise titration protocol for oral appliance therapy in positional obstructive sleep apnea patients: proof of concept

**DOI:** 10.1007/s11325-020-02045-w

**Published:** 2020-03-11

**Authors:** M. H. T. de Ruiter, G. Aarab, N. de Vries, F. Lobbezoo, J. de Lange

**Affiliations:** 1 Department of Oral and Maxillofacial Surgery of the Amsterdam UMC, University of Amsterdam, Meibergdreef 9, 1105 AZ Amsterdam, the Netherlands; 2 Department of Orofacial Pain and Dysfunction of the Academic Centre for Dentistry Amsterdam (ACTA), University of Amsterdam and Vrije Universiteit Amsterdam, Amsterdam, the Netherlands; 3 Department of Otorhinolaryngology and Head and Neck surgery, OLVG West, Amsterdam, the Netherlands; 4 Department of Otorhinolaryngology, Head and Neck Surgery Antwerp University Hospital, Antwerp, Belgium

**Keywords:** Oral appliance therapy, Mandibular advancement device, Obstructive sleep apnea, Sleep-disordered breathing, Titration protocol, Protrusion

## Abstract

**Purpose:**

In patients with positional obstructive sleep apnea (POSA), oral appliance therapy (OAT) is among the first-line treatments. The aim of this study was to evaluate the effects of a new standardized stepwise titration protocol for OAT in a group of patients with POSA.

**Methods:**

This was an observational intervention trial. Patients who were previously randomized to the OAT intervention arm of a comparison study comprised the subjects for this study. These patients, who had mild to moderate POSA, were assessed after 3 and 12 months for treatment efficacy, objective adherence by temperature microsensor, and side effects. The titration of OAT was performed using a standardized stepwise titration protocol including advancement levels of 60%, 75%, and 90% of the maximum mandibular protrusion. The optimal advancement level per individual was based on a weighted compromise between efficacy and side effects.

**Results:**

In total, 36 patients were included and all completed the titration protocol after 3 months. At baseline, the OAT was set at 60% of the maximal mandibular protrusion position. At a 3-month evaluation, the advancement remained at 60% in 16 patients (44%) and reached 75% advancement in 20 patients (56%). Mean apnea-hypopnea index decreased from 12.9 events per hour (9.1–16.7) to 6.9 (3.7–10.3) (*P* < 0.001), and median objective adherence was 97.4 (61.4–100.00) after 3 months. The 12-month analysis showed consistent results and good OAT tolerance. Six patients (16.7%) terminated OAT and one patient (2.8%) was lost to follow-up.

**Conclusions:**

This standardized stepwise titration protocol for OAT showed good efficacy, good OAT tolerance, and good objective adherence in patients with mild to moderate POSA. Therefore, the protocol is recommended in research projects to improve standardization of methods between studies and in clinical practice for its practical feasibility.

**Electronic supplementary material:**

The online version of this article (10.1007/s11325-020-02045-w) contains supplementary material, which is available to authorized users.

## Introduction

Obstructive sleep apnea (OSA) is the most common sleep-related breathing disorder. Overall prevalence is estimated from 9 to 38% in the general adult population, is higher in men, and rises with increasing age [1, 2]. Adequate treatment for OSA is indicated, as to counter problems in daily functioning, reduce cardiovascular and cerebrovascular risk, and sometimes even reduce mortality risk in severe OSA patients [3–5]. In patients with moderate to severe OSA, continuous positive airway pressure (CPAP) is the gold standard therapy [6]. Unfortunately, CPAP is not always well tolerated, which results in a suboptimal level of adherence [7]. Other treatment options are upper airway surgery, maxillomandibular advancement surgery, hypoglossal nerve stimulation, and oral appliances (OAs) [8, 9].

Positional OSA (POSA) is defined as an apnea-hypopnea index (AHI) that is at least twice as high in the supine position compared with that in the non-supine positions [10]. Prevalence is estimated at around 56% in mild OSA. In an additional 30% of the patients, the apneic events are higher in supine than in non-supine position, not reaching 50% difference [11]. For patients with mild to moderate POSA, oral appliance therapy (OAT) is among the first-line treatments, while positional therapy and surgery can be considered as well [12, 13]. OAs are widely used and often result in decreased AHI and oxygen desaturations (ODIs) with clinical improvement in excessive daytime sleepiness and snoring [14]. When OAs are compared with CPAP, the latter is in general more effective in reducing AHI [15]. However, OAs often have better adherence, which makes that CPAP and OAs have similar overall therapeutic effectiveness [16, 17].

Concepts about custom-made OAs have evolved from the monobloc OA towards a duobloc OA that consists of an upper and lower splint, which can be dynamically positioned against each other. During the titration procedure, the mandible is gradually positioned in a more anterior position to achieve a maximum therapeutic effect on opening the upper airway [18]. Equally important is to achieve a well-tolerated position, in order to ensure optimal compliance. Extensive evidence is available for the side effects of OAT, especially on teeth and the temporomandibular joints [19, 20]. It is therefore of utmost importance that the “target” protrusion of the mandible (1) is most effective on subjective (complaints) and objective (polysomnographic) metrics (AHI and ODI); (2) results in acceptable side effects in the short and long term; and (3) gives the highest possible adherence rate [21]. Currently, there is no consensus on the titration procedure, which makes it difficult to compare the outcomes of studies on OA and thus, there is a need for standardization. Therefore, the aim of this study was to determine the effects of a standardized titration protocol on the efficacy (i.e., decrease in AHI and ODI), self-reported side effects, and objective adherence in mild to moderate POSA patients in a 1-year follow-up.

## Materials and method

### Participants

This study is part of a randomized controlled trial in which OAT was compared with a position trainer in patients with POSA [12, 13]. Participants were eligible for enrollment if they had mild or moderate POSA, were 18 years or above, and were able to provide informed consent. Exclusion criteria were inadequate dentition for wearing an OA, subjective snoring in lateral position, diagnosed with central sleep apnea, night or rotating shift work, severe chronic heart disease, active psychiatric disease, seizure disorder, medication usage for sleeping disorders, muscular or joint injuries in head/neck or back area, previous OAT usage, simultaneous other treatments for OSA, reversible morphological upper airway abnormalities (e.g., enlarged tonsils), pregnancy, and coexisting non-respiratory sleep disorders (e.g., insomnia, periodic limb movement disorder, narcolepsy) that would compromise functional sleep assessment.

### Study design and oversight

This study is a sub-assessment of the OAT therapy group in the POSA trial [12, 13], focusing on the standardized *stepwise* titration protocol used. The POSA trial is a multicenter, prospective randomized controlled trial. The randomization sequence was generated by an independent clinical research unit. Allocation of treatment of 1:1 was performed with random block sizes of maximum 6 and stratified for smoking and body mass index (BMI). The protocol of this study was approved by the medical ethics committee (Amsterdam UMC: METC2012_208) and was registered before the start in clinicaltrials.gov (NCT02045576). All participants provided written informed consent before enrollment. Independent monitors performed verification of the source data and documentation. The study investigators had full access to the data and had the right to submit the manuscript for publication without input from the sponsor.

### Treatment

The OA was a custom-made duobloc (SomnoDent flex, SomnoMed, Sidney, Australia). The OA was adjusted individually, and advancement was titrated using a standardized *stepwise* titration protocol, developed by one of the authors (GA). After adequate assessment of the central relation and maximum protrusion using the George Gauge system with a standard 5-mm vertical dimension (Great Lakes Orthodontics, Tonawanda, NY), the OA was set at 60% advancement of maximum protrusion at baseline. During the first 3 months, at each consecutive visit, the OA was evaluated and advanced to 75% or 90% if subjective improvement (e.g., perceived reduction of snoring or apneic events) of OSA was not reached. On the other hand, if side effects were not acceptable for the patient (e.g., tooth pain or signs of temporomandibular disorders), the advancement was adjusted backward to 75%, 60%, or 45%. No adjustments were made when the patient reported a sufficient efficacy without side effects. The patient returned to clinical practice at 6, 10, and 14 weeks after placement of the OA for this standardized titration protocol. After the titration procedure was completed for each patient, they only returned when further adjustments were necessary, based on subjective impairments, viz., recurrent snoring or increasing excessive daytime sleepiness. The vertical dimension was not controlled in our patients using frontal elastics. Objective compliance was measured using a temperature-sensitive microsensor with on-chip integrated read-out electronics (Theramon, Handels- und Entwicklungsgeselschaft, Handelsagentur Gschladt, Hargelsberg, Austria). The temperature was recorded at a sampling rate of 1 measurement per 15 min, allowing data acquisition on usage for a consecutive 100-day period. A recorded temperature of > 30 °C indicated that the OA was worn. This microsensor was embedded in the OA at the lower right side. Data was extracted at 3 months (± 2 weeks) using a dedicated reading station. Missing results in the adherence data are due to problems with the reading station of the microsensors. Our titration protocol is enclosed as supplementary material 1.

### Outcome measures

The titration protocol was analyzed using different outcome measures. Polysomnographic response is presented by the AHI and ODI, and these parameters were used for assessment of therapy failure/success for OAT. Other measures were defined as patient’s adherence to OA treatment and self-reported side effects or discomfort resulting in termination of treatment. Adherence was defined as the percentage of daily use of ≥ 4 h per night, during ≥ 5 nights per week. Adherence failure was defined as an inability of the patient to continue treatment for any reason mentioned by the patient. These outcomes were recorded during the adaptation period for each new position of the mandible. Adverse events were reported in accordance with the International Conference of Harmonization ICH E2A guidelines (Good Clinical Practices) by the principal investigators and evaluated by clinical data monitors.

### Statistical analysis

Data were assessed on normality, both graphically using histogram plots and by the Shapiro-Wilk test and were analyzed and expressed as median (interquartile range) or mean ± SD for descriptive purposes. The presented variables were tested for differences using the Fisher exact test for categorical variables and the Wilcoxon signed-rank test for continuous variables with a non-normal distribution. When comparing groups between specific titration positions, the Kruskal-Wallis test was used in case of a non-normal distribution as an alternative for the ANOVA for normally distributed variables. Associations between continuous variables were described using Spearman’s Rho correlation. A *P* value < 0.05 was considered statistically significant. The test used for the statistical analysis is mentioned in the manuscript when a *P* value is provided (*T*, *t* test; *W*, Wilcoxon signed-rank test; and *H*, Kruskal-Wallis test).

## Results

**Fig. 1:**
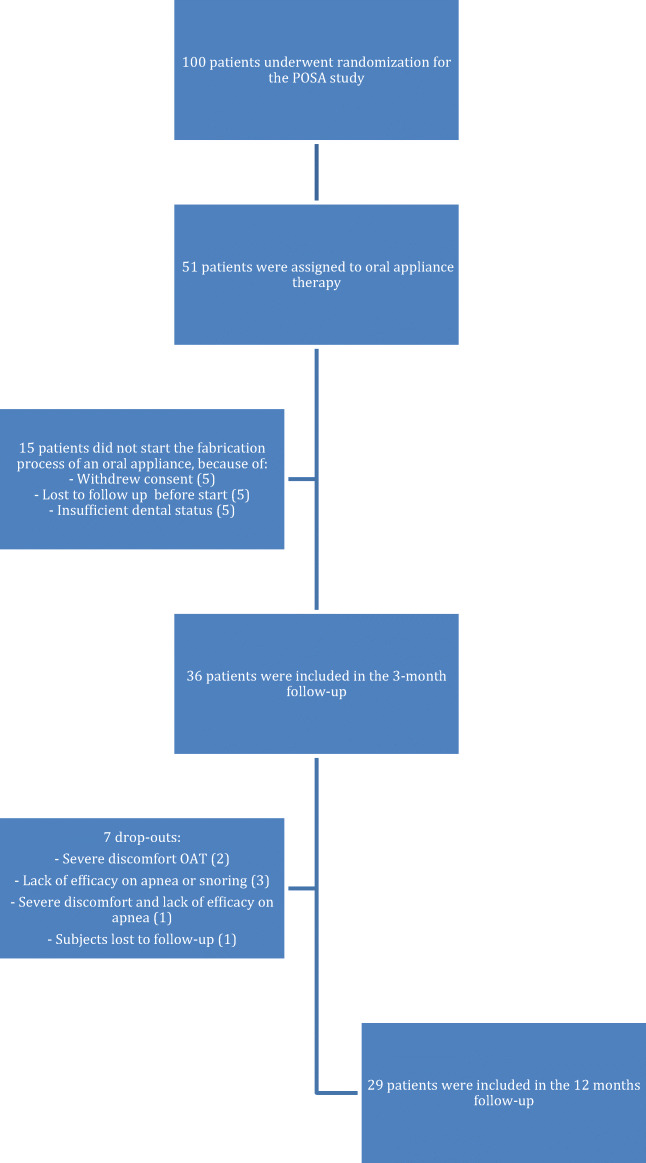
Flowchart of enrolled patients and dropouts

A total of 36 patients were included and all completed the titration protocol after 3 months. Six patients (16.7%) terminated OAT between 3 and 12 because of treatment-related reasons, and one patient (2.8%) was lost to follow-up (Fig. 1). This study cohort showed a mean ± SD age of 50.0 ± 9.4 years, 25 patients were male (69%), and mean BMI was 27.5 ± 3.8 kg/m^2^. At baseline, the AHI was 12.9 (9.1–16.7) events/h of sleep and ODI was 10.0 (6.0–13.8) events/h of sleep (Table 1). BMI of the patients increased slightly after 3 and 12 months from 27.5 to resp. 27.9 (*T* − 2.41; *P* = 0.021) and 28.9 (*T*, *−* 2.790; *P* = 0.009).

**Table 1:** Characteristics of the patients participated in the study

Variable (*n* = 36)	Value
Male, *n* (%)	25 (69)
Age, year (mean ± SD)	50,0 (± 9.4)
Body mass index, kg/m^2^ (mean ± SD)	27.5 (± 3.8)
Smoking, *n* (%)	7 (19)
Apnoea‑Hypopnea Index (median, (IQR 25–75))	12.9 (9.1–16.7)
Desaturation index (median, (IQR 25–75))	10.0 (6.0–13.8)
Epworth Sleeping Scale (mean ± SD)	8.1 (± 5.4)^a^

Data is presented with mean ± SD when it was normally distributed. When non-normally distributed as median (IQR 25–75)

^a^Data is based on 31 patients, because of missing data

After 3 months, 16 patients (44%) had their OA set at a 60% advancement level, while 20 of the OAs (56%) were set on an advancement level of 75%. The first evaluation of efficacy after 3 months by polysomnography with the OA in situ showed a statistically significant improvement in AHI; baseline AHI of 12.9 (9.1–16.7) events/h decreased after 3 months to 6.9 (3.7–10.3) events/h (*W*, 5.388; *P* < 0.001). When the protrusion level was evaluated after 3 months, the largest decrease in AHI and ODI was seen in patients set at an advancement level of 75%, which tended towards significance (*H*, 3.778; *P* = 0.052). None of the patients was titrated to 90% at the 3-month evaluation (Table 2).

**Table 2:** Overview of the polysomnographic characteristics of the study patients (*n* = 36) per titration position of OA

	Titration, 60%	Titration, 75%	Titration, 90%	*P* value
Baseline (*n* = 36)	(*N* = 36)			
AHI ODI	12.9 (9.1–16.7) 10.0 (6.0–1.8)	- -	- -	
3-month follow-up (*n* = 36)	(*N* = 13)	(*N* = 23)	-	0.052
AHI ODI	9.7 (5.1–14.8) 8.0 (4.0–12.0)	5.0 (3.4–9.4) 5.0 (3.0–8.0)	-	0.159
12-month follow-up (*n* = 29)	(*N* = 3)	(*N* = 15)	(*N* = 11)	
AHI ODI	5.4 (5.0–5.4) 7.0 (6.0–7.0)	4.4 (2.9–6.6) 4.0 (2.0–8.0)	7.8 (4.0–14.7) 8.0 (7.0–16.0)	0.127 0.052

All patients started at baseline on 60% of maximum protrusive path

The table gives an overview of median (IQR25–75)

Differences between groups are significant (Kruskal-Wallis test *P* < 0.05)

At long-term evaluation (12 months), only 3 patients (8.3%) were still on 60% advancement, 41.7% (15 patients) were in 75% protrusion, and 30.6% (11 patients) of the OAs were set in a 90% advancement position. The AHI after 12 months decreased significantly to 5.0 (3.9–8.9) events/h compared with baseline AHI (*W*, 5.073; *P* < 0.001). Between 3 and 12 months, there was no significant difference in AHI (*W*, 0.492; *P* = 0.531). At 12 months, patients showed a trend towards the highest efficacy in the 75% advancement group compared with the 60% and 90% groups (*H*, 5.928; *P* = 0.052).

The different success definitions with their respective percentages are summarized in Table 3. Treatment success defined as an AHI below 5 events per hour was achieved in 41.7% of the patients after 3 months, and in 51.7% after 12 months. Treatment success defined as an AHI decrease of more than 50% was seen in 55.2% of the patients after 12 months. If treatment success was defined by an AHI below 10, the population showed 75% treatment success after 3 months and almost 80% success after 12 months.

**Table 3:** Treatment success definitions of the present study

Success definition	Percentage (%) at 3 months (*N* = 36)	Percentage (%) at 12 months (*N* = 29)
Treatment response (decrease of ≥ 50% in AHI)	47.2	55.2
Treatment success (AHI < 5/h)	41.7	51,7
Treatment success (AHI < 10/h)	75.0	79.3
Decrease of ≥ 50% in AHI and AHI < 5/h	30.6	41.4
Decrease of ≥ 50% in AHI and AHI < 10/h	44.4	55.2
Adherence failure	0	17.1

A correlation analysis showed no significant correlation between AHI decreased after 3 and 12 months in comparison with the mandibular protrusion levels that increased in this specific period, viz., 0.07 (*P* = 0.687) and 0.14 (*P* = 0.476), respectively.

### Side effects of treatment

All patients completed the titration protocol in the first 3 months. None of the patients ended treatment in the first 3 months because of discomfort or side effects due to OAT. Two patients encountered severe problems with OAT, for which titration was set back to 45% in the beginning, but at the evaluation period at 3 months with PSG, the titration was set again at 60%, after symptoms improved. One of these two patients had no decrease of AHI together with severe discomfort while wearing OA and terminated the study directly after the 3-month evaluation. The other patient could be motivated to continue therapy and eventually ended the study at 12 months successfully on a titration of 75% with good efficacy. After the 3-month evaluation with PSG, two patients terminated OAT because of severe discomfort in wearing the appliance, and three patients ended the study preliminary because of lack in efficacy with no desire to continue the protocol (no decrease AHI and persistent excessive daytime sleepiness). These patients did not report any discomfort. In total, six patients (17%) terminated OAT because of treatment-related reasons. One patient was lost to follow-up at 9 months because of emigration.

### Adherence

**Fig. 2:**
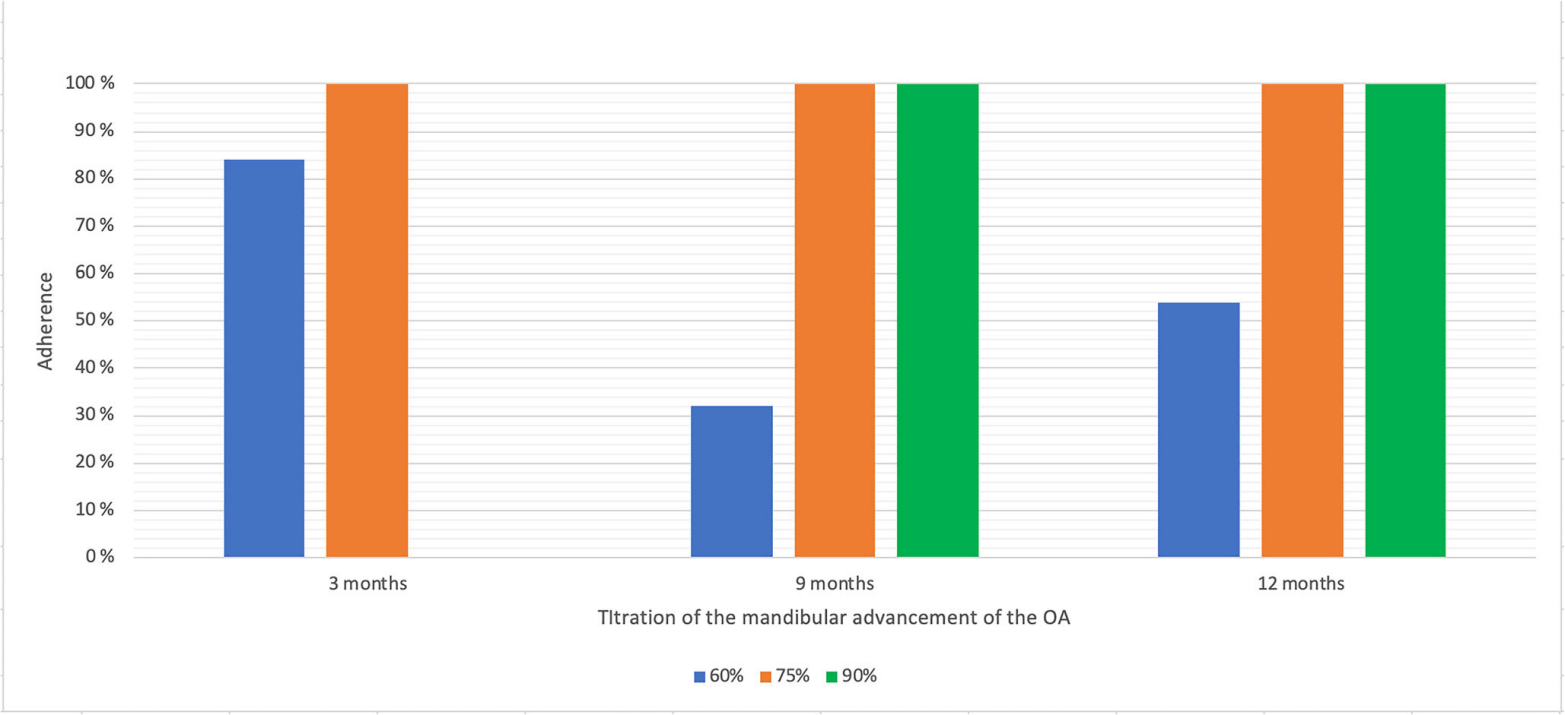
Overview of the adherence data of the study patients per titration position of OA

Median adherence (> 4 h, 5 nights a week) was 97.4% (61.4–100.00) after 3 months of OAT (*N* = 33) and 100.0% (90.0–100.0) after 12 months (*N* = 23). No differences were seen after 3 months between the different advancement levels (*H*, 2.567; *P* = 0.109). After 9 and 12 months of OA use, the median adherence was excellent (100%) in the 75% and the 90% titration groups. The few patients (*n* = 2 and *n* = 3) in the 60% titration group after 9 and 12 months showed lower adherence when compared with the 75% and the 90% groups (*H*, 1.122; *P =* 0.571) (Fig. 2 and Table 4).

**Table 4:** Overview of the adherence data of the study patients per titration position of OA

	Titration, 60%	Titration, 75%	Titration, 90%	*P* value
Adherence 3 months 5 of 7 days	(*n* = 11) 83.7 (50.4–100.0)	(*n* = 22) 100.0 (70.5–100.0)	–	0.109
Adherence 9 months 5 of 7 days	(*n* = 3) 32.3 (18.6– -)	(*n* = 14) 100.0 (64.2–100.0)	(*n* = 9) 100.0 (100.0–100.0)	0.082
Adherence 12 months 5 of 7 days	(*n* = 2) 54,3 (8.6– -)	(*n* = 11) 100.0 (80.0–100.0)	(*n* = 10) 100.0 (91.1–100.0)	0.571

All patients started at baseline on 60% of maximum protrusive path. Missing data is due to chip information processing problems

The table gives an overview of median (IQR 25–75)

Differences between groups are significant (Kruskal-Wallis *P* < 0.05)

## Discussion

This is the first prospective study that evaluated a standardized step wise titration protocol for OAT in patients with mild and moderate POSA. It showed that this titration protocol is effective in treating patients by significantly reducing AHI and ODI in a short- and long-term analysis. The rate of adherence was high for the different advancement levels. After 3 months of treatment, no patients terminated the study, while after 12 months, six patients (17%) stopped the treatment because of adverse effects.

The treatment success of this protocol is in accordance with results available in the literature. Our treatment success (AHI decrease of ≥ 50%) is 50–55%, while in the literature, 40–65% is reported [16, 22–25]. It is as yet unknown whether OAT is more or less effective in supine-positioned OSA. Marklund et al. showed in a cohort of 26 patients that an OA would be more effective in supine-positioned OSA in comparison with that in non-positional OSA [24]. Chung et al. confirmed this hypothesis and concluded that the OAT is better in lowering AHI in position-dependent OSA compared with non-positional OSA [23]. However, more recently, a large retrospective cohort study of Sutherland et al. showed better results of OAT in non-positional OSA compared with patients with positional OSA, 36% vs. 59%, for an AHI below 10 [25]. When applying the definition of treatment success according to Sutherland et al. (AHI below 10), our success percentage was 75% after 3 months and almost 80% after 12 months. Another large cohort in non-positional patients showed a mean outcome of 52% [22]. For treatment cure (success defined as an AHI decrease below 5 events per hour), our protocol achieved success in 41.7% after 3 months and in 51.7% after 12 months. Ferguson et al. found an average of 42% applying the same definition of success [22]. The different treatment outcomes between the above-cited studies and our study could be explained by patient selection or different titration protocols. Phenotyping can identify specific patient characteristics, e.g., BMI, neck circumference, age, and baseline AHI values. In addition, most of the above-cited studies do not report the specific titration protocol that was used. More recent study of Milano et al. showed the importance of controlling the vertical dimension of the OAT, especially in patients with POSA [26]. The OA with elastic fixation is significantly better in treating patients with POSA. In our study, none of the patients received elastic retention.

The relatively good outcome of our study can be contributed to the fact that in this protocol, the treating dentist was adjusting the OA to its most optimal protrusive position in several visits based on a combination of efficacy, subjective parameters, and objective adherence. In our population, the group of patients with an advancement of 75% had the most favorable outcome after 3 and 12 months compared with the other groups (60% and 90% advancement), yet this result showed no significance, possibly due to the small sample size with a post hoc power of 47.5%. Power calculation using our data shows a preferred group sample size of *n* = 39 per group to yield a power of 90. It can be reasoned that most patients will receive optimal treatment in 75% advancement, while in case of insufficient improvement, advancement to 90% can be considered. Available studies show an effective response on AHI already at a protrusion of 25%, but more response is achieved when the advancement is increased to 50% or 75% [27, 28]. Thereby, our results show that 90% advancement is not always yielding the most effect on AHI. Our cohort showed an optimal position of 75% advancement in most patients. It could be advocated that further protrusion to 90% should only be considered in case of insufficient improvement with 75% protrusion and in case further protrusion is not contraindicated because of side effects [16].

No correlation was found in our cohort between the decrease in AHI and amount of mandibular protrusion. Therefore, every titration is based probably on other (yet unknown) individual characteristics and not mandibular protrusion.

This is the first study wherein objective adherence was assessed in relation to mandibular protrusion levels as dictated by efficacy and side effects. A limited number of studies report objective adherence data unrelated to a specific titration protocol; overall mean outcome of adherence in OAT was around 80% [29]. Our study shows similar outcome data of adherence at our different advancement levels. At 12 months, the adherence is better in the patients of the 75% and 90% groups compared with those in the 60% group; in other words, a better adherence is realized with more protrusion of the mandible. A possible explanation for this phenomenon could be that these patients are ambivalent to negative outcomes of the OAT (e.g., side effects) and are more motivated to reach an optimal treatment. On the other hand, patients who declined more advancement in their OA were less adherent.

Our study has some notable limitations. Although this study is part of an RCT, the proposed titration protocol was not part of the initial research question. Therefore, this study lacks a control group and so this article demonstrates the *proof of concept* of our titration protocol rather than an analysis on possible superiority or non-inferiority. The review of Dieltjens et al. describes several titration protocols based on subjective, objective, or a combination of both outcomes and concludes that at present, no gold standard titration protocol is available, and the titration of the OAT is based on “trial and error” procedure in daily clinical practice [18]. For future research, we propose using our standard *stepwise* titration protocol, so that different outcomes in subjective and objective improvement with the OAT can be evaluated using standardized outcome measures. Possible negative effects of OAT might be attributed to a suboptimal adjustment of the OA. This will also enhance comparability of research projects on OAT and provide insight into other variables that might influence the efficacy of OAT, like e.g. specific jaw dysmorphology or the vertical dimension of the OA [27].

## Conclusion

This standardized stepwise titration protocol for OAT showed good efficacy, good OAT tolerance, and good objective adherence in mild to moderate POSA. Therefore, the protocol is recommended in research projects to improve the comparability between studies and in clinical practice for its practical feasibility.

## Electronic supplementary material

ESM 1 (DOCX 17 kb)

ESM 2 (DOCX 17 kb)
