# Supplementary material for: A stepwise titration protocol for oral appliance therapy in positional obstructive sleep apnea patients: proof of concept
Source: Sleep Breath. 2020 Mar 11;24(3):1229–36. doi: 10.1007/s11325-020-02045-w (PMC7426292; doi:10.1007/s11325-020-02045-w)
Supplement: Supplementary file 1 — (DOCX 17 kb) [file 11325_2020_2045_MOESM1_ESM.docx]

**Supplementary materials**

**Table 1s.**

| **Table 1s.** | **Central Relation** | **Maximum Protrusion** | **Total protrusive path** | **Path 60%** | **Therapeutic position** |
| --- | --- | --- | --- | --- | --- |
| Mean | -6.4 | 5.5 | 12.0 | 7.2 | 0.8 |
| Median | -6.0 | 5.0 | 12.0 | 7.0 | 1.0 |
| Minimum | -10.0 | 2.0 | 7.0 | 4.0 | -2.0 |
| Maximum | -4.0 | 10.0 | 16.0 | 10.0 | 4.5 |
