# Supplementary material for: A stepwise titration protocol for oral appliance therapy in positional obstructive sleep apnea patients: proof of concept
Source: Sleep Breath. 2020 Mar 11;24(3):1229–36. doi: 10.1007/s11325-020-02045-w (PMC7426292; doi:10.1007/s11325-020-02045-w)
Supplement: Supplementary file 2 — (DOCX 17 kb) [file 11325_2020_2045_MOESM2_ESM.docx]

**Supplementary materials.**

**Table 2s.**

| **Table 2s.** | **Baseline** | **3 months** | **Extra visit** | **12 months** |
| --- | --- | --- | --- | --- |
| Protrusion 45% | - | - | - | - |
| Protrusion 60% | 100 | 44.4 | 11.8 | 10.3 |
| Protrusion 75% | - | 55.6 | 52.9 | 51.7 |
| Protrusion 90% | - | - | 35.3 | 37.9 |
